# Histone Deacetylase Inhibitor Modulates NKG2D Receptor Expression and Memory Phenotype of Human Gamma/Delta T Cells Upon Interaction With Tumor Cells

**DOI:** 10.3389/fimmu.2019.00569

**Published:** 2019-03-27

**Authors:** Jaydeep Bhat, Samuel Dubin, Alexandra Dananberg, Elgar Susanne Quabius, Juergen Fritsch, C. Marie Dowds, Ankit Saxena, Guranda Chitadze, Marcus Lettau, Dieter Kabelitz

**Affiliations:** ^1^Institute of Immunology, University Hospital Schleswig-Holstein, Kiel, Germany; ^2^Department of Oto-Rhino-Laryngology, University Hospital Schleswig-Holstein, Kiel, Germany; ^3^Institute of Clinical Molecular Biology, Kiel University, Kiel, Germany; ^4^National Heart, Lung and Blood Institute, National Institutes of Health, Bethesda, MD, United States

**Keywords:** gamma/delta T cells, HDAC inhibitor(s), histone acetylation, NKG2D, memory T cells, tumor microenvironment, valproic acid

## Abstract

The functional plasticity and anti-tumor potential of human γδ T cells have been widely studied. However, the epigenetic regulation of γδ T-cell/tumor cell interactions has been poorly investigated. In the present study, we show that treatment with the histone deacetylase inhibitor Valproic acid (VPA) significantly enhanced the expression and/or release of the NKG2D ligands MICA, MICB and ULBP-2, but not ULBP-1 in the pancreatic carcinoma cell line Panc89 and the prostate carcinoma cell line PC-3. Under *in vitro* tumor co-culture conditions, the expression of full length and the truncated form of the NKG2D receptor in γδ T cells was significantly downregulated. Furthermore, using a newly established flow cytometry-based method to analyze histone acetylation (H3K9ac) in γδ T cells, we showed constitutive H3K9ac^low^ and inducible H3K9ac^high^ expression in Vδ2 T cells. The detailed analysis of H3K9ac^low^ Vδ2 T cells revealed a significant reversion of T_EMRA_ to T_EM_ phenotype during *in vitro* co-culture with pancreatic ductal adenocarcinoma cells. Our study uncovers novel mechanisms of how epigenetic modifiers modulate γδ T-cell differentiation during interaction with tumor cells. This information is important when considering combination therapy of VPA with the γδ T-cell-based immunotherapy for the treatment of certain types of cancer.

## Introduction

Immune cells have the capacity to recognize self-antigens which are upregulated in response to viral infection, DNA damage, or cellular transformation. NKG2D (natural-killer group 2, member D) is one of the receptors recognizing such upregulated self-proteins. NKG2D is a C-type lectin-like type II transmembrane glycoprotein, expressed on almost all human NK cells and γδ T cells, substantial proportions of NKT cells and, CD8 T cells, and a small subset of CD4 T cells (1). Human NKG2D is an activating receptor that recognizes two families of ligands. The first family of NKG2D ligands includes the highly polymorphic MHC class I chain-related A (MICA) and B (MICB) proteins, while another family comprises the UL16-binding proteins (ULBP1-6). The NKG2D receptor/ligand interaction plays an important role in regulating the γδ T-cell mediated cytotoxicity against a broad range of tumor cells (2, 3) and NK cell activity (4). In order to escape immune cell attack, tumor cells can release NKG2D ligands from the cell surface by proteolytic cleavage (shedding) (5). Shedding of NKG2D ligands varies among tumor entities and can involve different enzymes like a-disintegrin-and-metalloprotease (ADAM) proteases and matrix metalloproteases (MMP) (6, 7). However, it is still under extensive investigation whether shedding of NKG2D ligands is a pro-tumorigenic or anti-tumor immune response (8, 9).

Many biological processes are regulated by epigenetic mechanisms. Dysregulation of such fundamental processes may lead to the development of cancer. Targeting epigenetic mechanisms including DNA methylation and histone modifications by various inhibitors or small molecules holds the potential for novel therapeutic approaches in oncology (10). Proteins involved in epigenetic regulation are divided into three distinct groups based on broad functions: “writers,” “erasers,” and “readers.” The most widely studied enzymes, DNA methyltransferases (DNMT) and histone acetyltransferases (HAT), are epigenetic writers, which set up epigenetic marks on DNA or associated histones. On the other hand, histone deacetylases (HDAC) are epigenetic erasers, which remove such marks (11). Pharmacological inhibitors specific for respective epigenetic enzymes have been identified as potential candidates for clinical application. The DNMT inhibitors Decitabine and Epigallocatechin-3-gallate (EGCG), the HAT inhibitor Curcumin and the HDAC inhibitors Valproic acid (VPA), Trichostatin A (TSA) and 4-Phenylbutric acid (4-PBA) are already in the clinic or in clinical trials for the treatment of various diseases (12–16).

In the present study, we show that an HDAC inhibitor significantly increases the expression and release of NKG2D ligands from pancreatic and prostate carcinoma cell lines. Specifically, the HDAC inhibitor VPA modulates NKG2D receptor gene and protein expression in γδ T cells upon co-culture with tumor cells. We established a flow cytometry-based method to analyze H3K9 acetylation in γδ T cells, which revealed substantial alterations in the subset distribution of memory γδ T cells as an effect of VPA treatment in co-cultures with tumor cells. Thus, we demonstrate that the functional plasticity of γδ T cells depending on H3K9 acetylation status is affected by *in vitro* tumor microenvironment and is additionally modulated by clinically approved epigenetic modifiers. These findings will help to optimize the clinical applicability of γδ T cells depending on the *in vitro* activity against distinct tumors.

## Results

### HDAC Inhibitors Differentially Modulate NKG2D Ligand Surface Expression and Release From Pancreatic Carcinoma and Prostate Carcinoma Cells

Previous findings from our group have shown that the pancreatic carcinoma cell line Panc89 is heterozygous for MICA^*^009:01 (A6) and MICA^*^027 (A5), and the prostate carcinoma cell line PC-3 is heterozygous for MICA^*^008:01:01 (A5.1) and MICA^*^012:01:01 (A4). Based on these differences of MICA alleles, Panc89 cells shed MICA/B by proteolytic cleavage, whereas PC-3 cells release MICA via exosomes (6). To address the potential role of epigenetic regulation in the mechanism of NKG2D ligand shedding, we used six different epigenetic inhibitors (Decitabine, EGCG, Curcumin, VPA, TSA, and 4-PBA) specific for different important epigenetic processes. The experimental strategy to investigate the effect of epigenetic inhibitors on Panc89 and PC-3 cells is schematically illustrated in Supplementary Figure 1. All epigenetic modifiers were titrated for their cell type dependent effective dose concentrations (data not shown) (17, 18).

**Figure 1:**
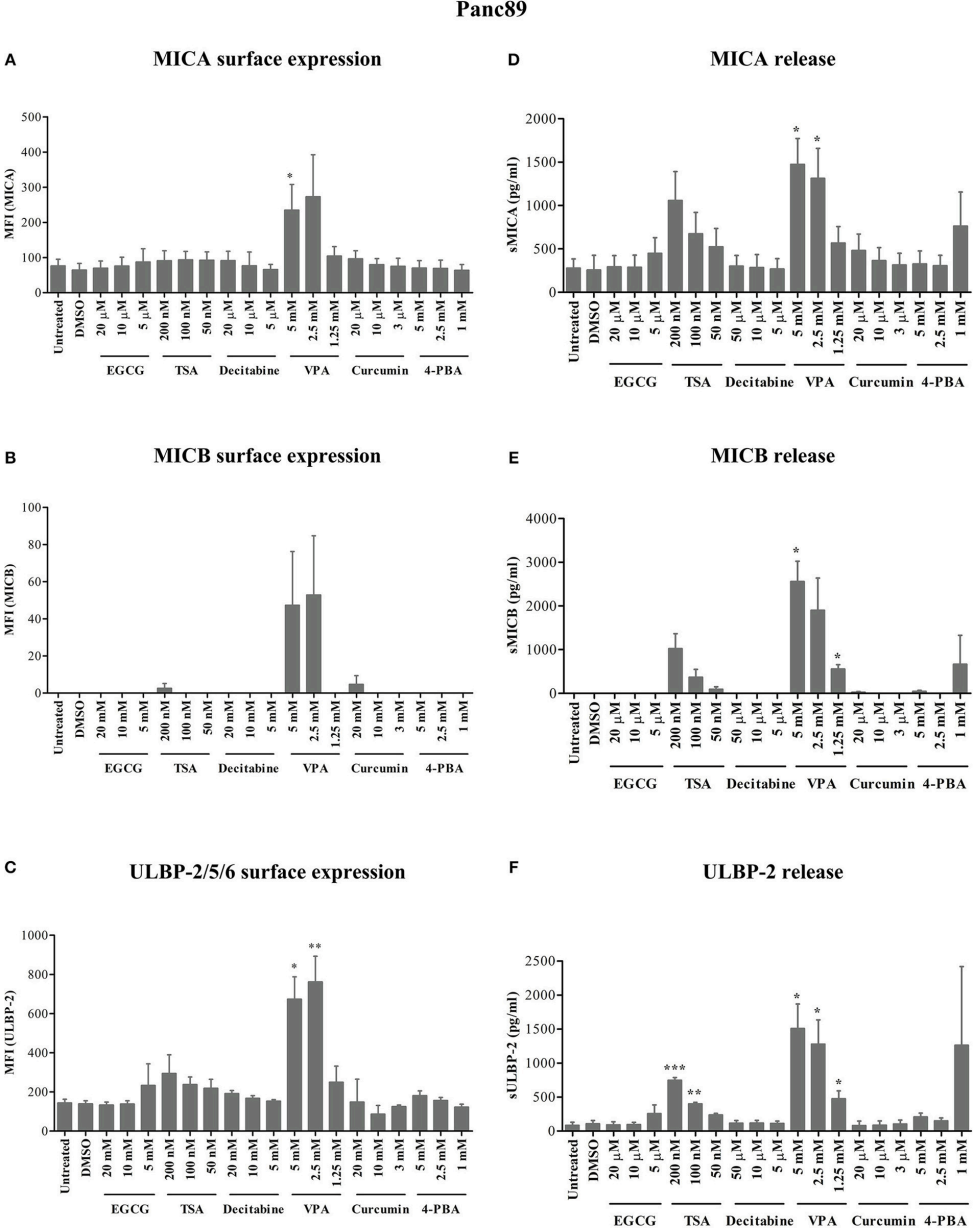
Modulation of NKG2D ligand expression and release from a pancreatic cancer cell line by epigenetic modifiers. As schematically shown in Supplementary Figure 1, 0.8 × 10^6^ Panc89 cells were treated with varying concentrations of inhibitors for HDACs, HATs and DNMTs. **(A–C)** After 24 h, cells were harvested for the analysis of MICA, MICB and ULBP-2/5/6 surface protein expression, respectively. **(D–F)** Culture supernatants from the same experiments were analyzed for the release of MICA, MICB, and ULBP-2 using respective ELISA. Data represents mean values ± S.E. of three independent experiments. Statistical significances with *p*-value ≤ 0.05, 0.01, and 0.005 in comparison to untreated or DMSO treated cells are indicated by ^*^, ^**^, or ^***^, respectively.

**Figure 2:**
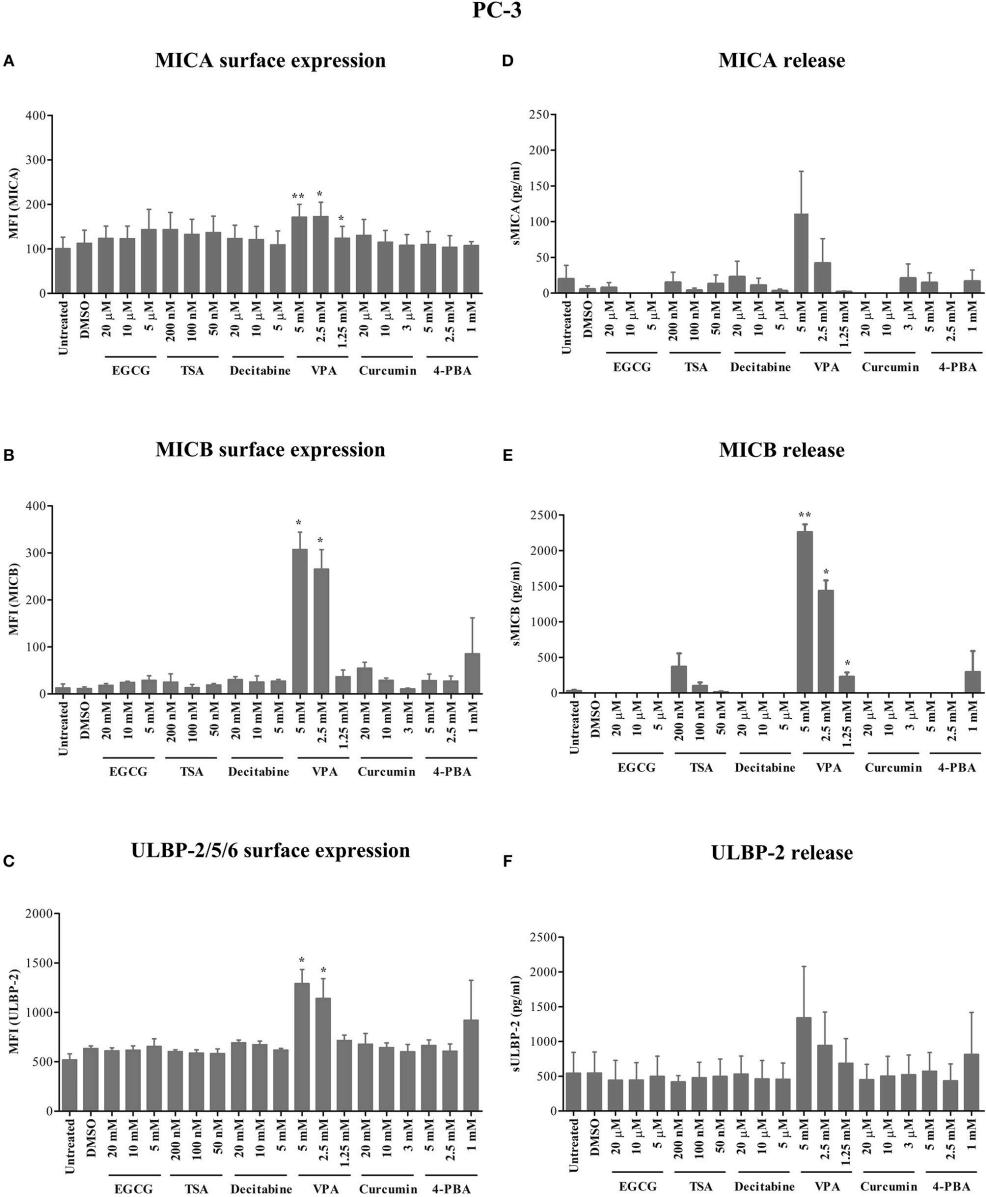
Modulation of NKG2D ligand expression and release from a prostate cancer cell line by epigenetic inhibitors**. (A–C)** In a similar approach as shown for Panc89 cells in Figure 1, PC-3 cells were analyzed for MICA, MICB and ULBP-2/5/6 surface expression. **(D–F)** Culture supernatants were quantitated for respective soluble NKG2D ligand proteins using ELISA. Data are summarized as mean ± S.E. from three independent experiments and *p*-values for <0.05 and 0.01 in comparison to untreated or DMSO treated cells are shown as ^*^, and ^**^, respectively.

After 24 h of treatment, VPA concentrations of 5 and 2.5 mM significantly increased ULBP-2/5/6 cell surface expression on Panc89 cells (Figures 1A–C). PC-3 cells also showed a strong and highly significant increase in the expression of MICB and ULBP-2/5/6, however the increase in MICA expression was only moderate but still significant after 5 mM and 2.5 mM VPA treatment (Figures 2A–C). Representative histograms of NKG2DL cell surface expression on Panc89 and PC-3 are shown in Supplementary Figure 2. Analysis of cell culture supernatants by ELISA also showed a remarkable increase in the release of soluble NKG2D ligands from both cell lines after treatment with 5 and 2.5 mM VPA (Figures 1D–F, 2D–F). In contrast, there was no increase in ULBP-1 cell surface expression and release from Panc89 and PC-3 cell lines upon treatment with epigenetic inhibitors (data not shown). Treatment with the HDAC inhibitor TSA also induced an increase in the release of MICA, MICB and ULBP-2 from Panc89 cell culture supernatants, but not in surface expression, and no effect was observed in PC-3 cells. Of note, the epigenetic modifiers did not induce notable cell death in the tumor cell lines at the concentration used (data not shown), in contrast to the effect observed on γδ T cells (17). Additionally, in a similar experimental set-up, a slight or no induction of surface NK2DL protein and/or release of NKG2DL from γδ T cells were observed (Supplementary Figure 3) reiterating the previously reported role of post-transcriptional regulation (19, 20).

Thus, out of six different epigenetic modifiers tested, only HDAC inhibitor VPA increased cell surface expression and/or release of MICA, MICB and ULBP-2 from the pancreatic carcinoma and prostate carcinoma cell lines irrespective of their allelic MICA variation.

### VPA Affects NKG2D Receptor Expression on the Cell Surface of Activated γδ T Cells Under *in vitro* Tumor Co-culture Conditions

**Figure 3:**
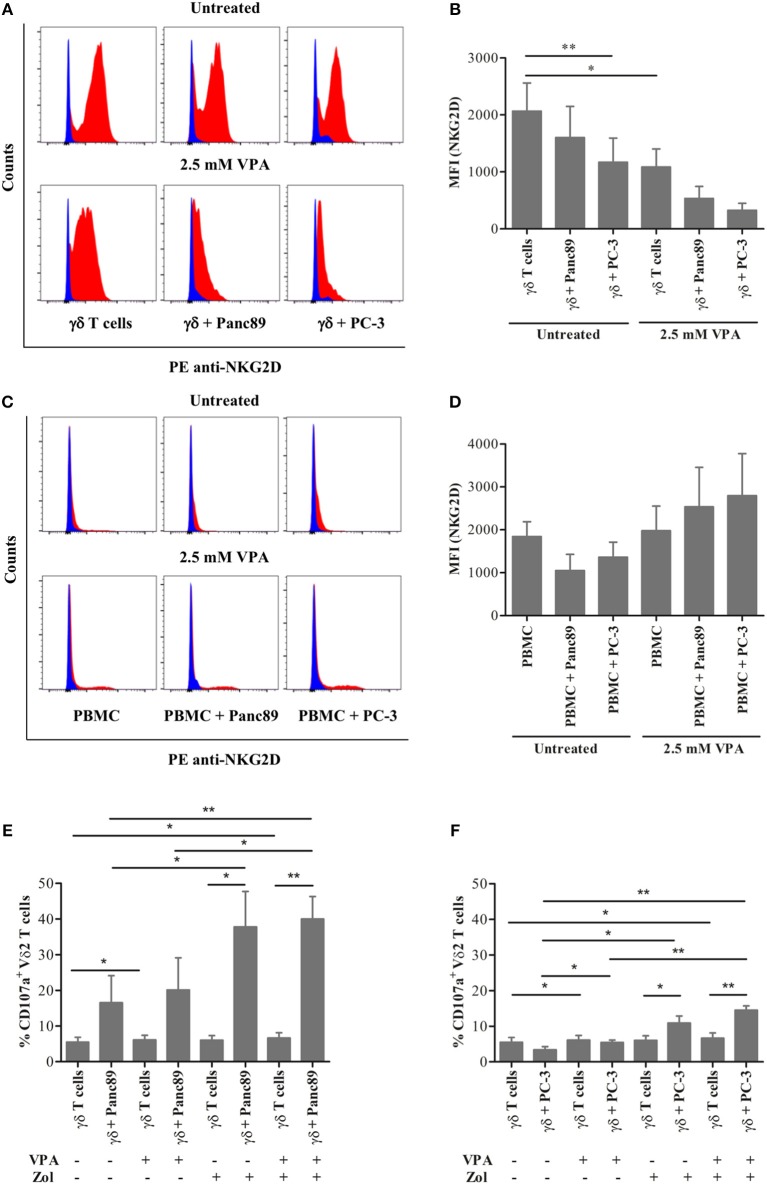
Effect of the HDAC inhibitor VPA on NKG2D receptor expression on effector cells. γδ T cells from 12 days zoledronic acid-stimulated PBMC **(A–B)** or freshly-isolated PBMC **(C–D)** were co-cultured with Panc89 and PC-3 cells at a 1:1 ratio in the presence or absence of 2.5 mM VPA, as described in Supplementary Figure 1. After 24 h of co-culture, γδ T cells **(A–B)** or PBMC **(C–D)** were collected by gentle pipetting and analyzed for NKG2D receptor expression by flow cytometry. In the representative histograms **(A,C)**, the upper panel represents the untreated effector cells or co-culture with Panc89 or PC-3 cells, and the lower panel represents additional 2.5 mM VPA treatment. Blue histograms represent isotype controls, while red histograms represent anti-human PE-NKG2D staining. A summary of three to four independent experiments as bar plots is represented by Median Fluorescence Intensity (MFI) in **(B,D)**. MFI was calculated by subtracting MFI of isotype controls from MFI of test antibodies. Mean values ± S.E. of median fluorescence intensity (MFI) in the bar plot are shown in comparison to untreated solo γδ T cells with statistically significant *p*-value < 0.01 or 0.05 as ^**^ or ^*^, respectively. In a similar set-up, γδ T cells were analyzed for the expression of CD107a (degranulation marker) after 24 h co-culture with **(E)** Panc89 and **(F)** PC-3 in the presence or absence of VPA and Zol (Zoledronate). The data represented here is the mean values ± S.E. of CD107a^+^ Vδ2 T cells from three independent experiments. The statistical significance is shown by ^**^ or ^*^ for *p*-value < 0.01 or 0.05, respectively. Vδ2 T cells or lymphocyte-gated PBMC were acquired after respective staining. The results were further analyzed using FlowJo software.

The previous experiments showed that VPA induces a significant increase in MICA/B and ULBP-2 surface expression and release from tumor cells of different origin. Using a co-culture experiment setting (see Supplementary Figure 1), we tested the effect of VPA-stimulated NKG2D ligand release on effector cells, i.e., freshly isolated PBMC or short-term γδ T-cell lines established from zoledronic acid-stimulated PBMC. The nitrogen-containing bisphosphonate zoledronic acid induces selective expansion of Vδ2 T cells due to the endogenous production of the γδ T-cell stimulating isopentenyl pyrophosphate (IPP) in the eukaryotic mevalonate pathway (21). As expected, γδ T cells down-modulated NKG2D receptor expression upon co-culture for 24 h with Panc89 and PC-3 cells (Figure 3A, upper panel). This effect was enhanced by VPA treatment of tumor cells for 24 h before co-culture (Figure 3A, lower panel). Corroborating our previous report (17), VPA treatment of γδ T cells significantly decreased NKG2D receptor expression. Interestingly, this effect was not observed in co-cultures with freshly isolated PBMC (Figure 3C, lower panel). A summary of three to four experiments is presented in Figure 3B (co-culture with γδ T cells) and Figure 3D (co-culture with PBMC). The selective downregulation of the NKG2D receptor on γδ T cells is of importance, specifically in the tumor co-culture, in addition to the dose-dependent effect of VPA treatment (17). Taken together, these results may support the previous notion that ligand binding induces down-modulation of NKG2D cell surface expression, which is further regulated by the HDAC inhibitor VPA during *in vitro* co-culture with tumor cells.

Cytotoxic effector activity of human γδ T cells against a broad range of tumor cells can be triggered via the T-cell receptor (TCR) or via the NKG2D receptor, and is known to be modulated by cell interaction molecules like lymphocyte function-associated antigen-1 (LFA-1) (2, 22). We analyzed degranulation (indicative of effector cell cytotoxicity) using the CD107a analysis within the same experimental set-up to study if decrease in NKG2D receptor expression on γδ T cells and/or increase in NKG2D ligand release by tumors affect the degranulation. The γδ T cells showed remarkable degranulation upon co-culture with Panc89 but not with PC-3 (Figures 3E,F). In line with our expectation, the CD107a expression was significantly enhanced after zoledronate treatment, but little additive/synergistic effect was observed upon VPA treatment. Given the obvious differences between co-cultures with Panc89 and PC-3 tumor cells (Figures 3E,F), it appears that the VPA-induced increase in NKG2D ligand expression and/or release from tumor cells and the decrease in NKG2D receptor expression on γδ T cells during co-culture does not directly reflect the level of cytotoxic activity of γδ T cells against tumor cells as revealed by the CD107a degranulation assay.

### VPA Regulates mRNA Expression of NKG2D Receptor and Its Ligands

**Figure 4:**
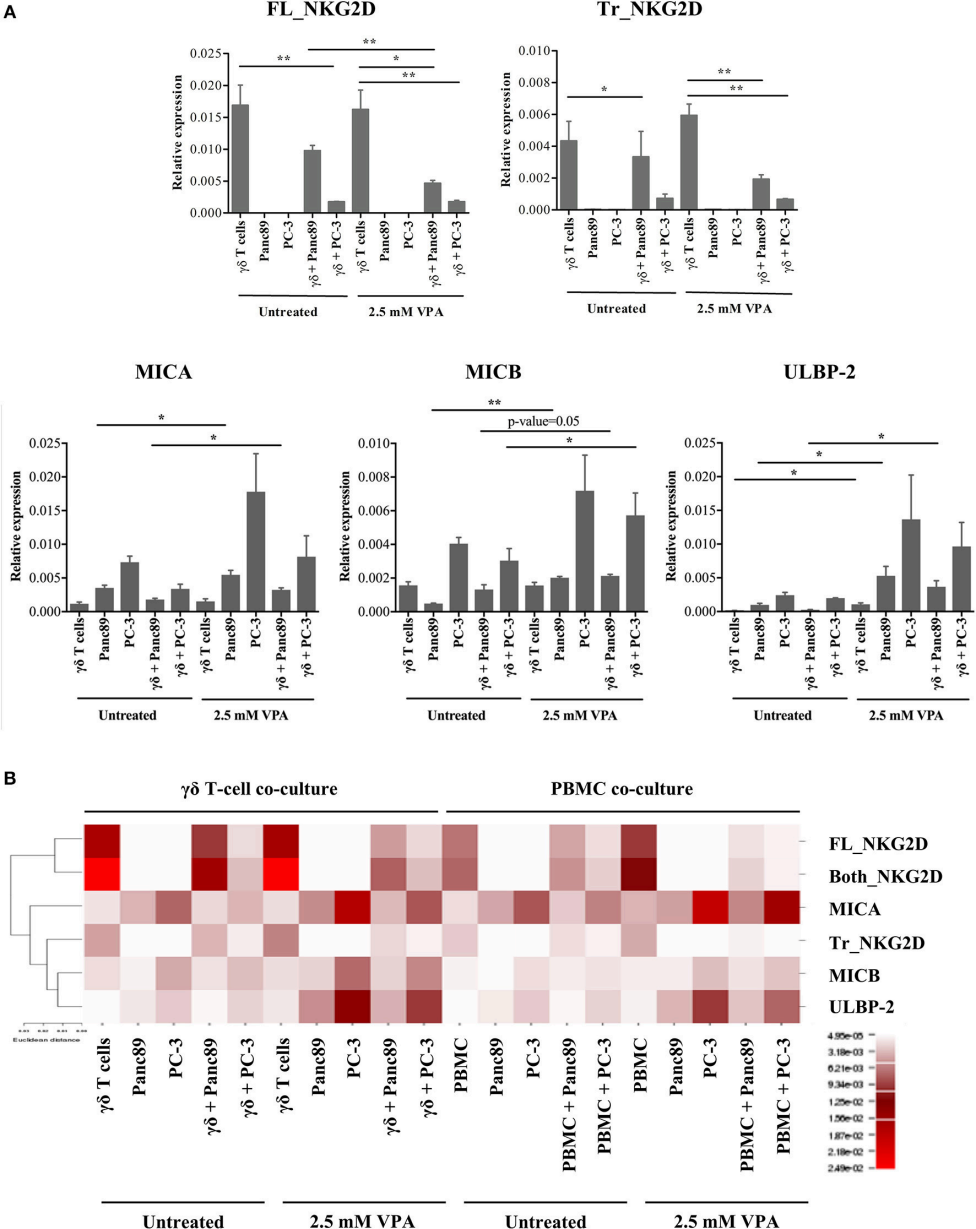
VPA-mediated regulation of NKG2D receptor and ligand gene expression. **(A)** γδ T cells were treated with 2.5 mM VPA or left untreated and co-cultured with or without Panc89 and PC-3 cells. After 24 h, γδ T cells from co-cultures and solo-culture, or adherent tumor cells from solo-cultures (as an internal control) were harvested and analyzed for mRNA expression of full length NKG2D (FL_NKG2D), truncated NKG2D (Tr_NKG2D), MICA, MICB, and ULBP-2/5/6. mRNA levels were calculated as relative expression values compared to the mean Ct value of the housekeeping genes (β-actin, β2-microglobulin and 18S rRNA). Graphs represent mean values ± S.E. of three independent experiments with *p*-values < 0.01, 0.05 as ^**^, ^*^. **(B)** Based on the relative expression level, the heatmap illustrates unsupervised hierarchical clustering of respective mRNA for NKG2D receptor-ligands using Euclidian distance method with average linkage rule. Though the graph of relative expression for sequences covering both the full length and truncated NKG2D gene (Both_NKG2D, as an experimental control which includes both truncated and full-length variants) is not shown in the Figure 4A, the expression values were used for the hierarchical clustering. The color and intensity of the boxes is used to represent changes of gene expression as indicated in the color legend.

Using overexpression systems and biochemical approaches, Karimi et al. recently showed that a truncated NKG2D isoform (referred hereafter as Tr_NKG2D) competitively interferes with the full-length form (referred hereafter as FL_NKG2D) resulting in decreased NKG2D cell surface expression (23). Since we observed a differential modulation of NKG2D receptor expression on the surface of γδ T cells and PBMC in the presence of VPA (Figure 3), we next determined the gene expression level of Tr_NKG2D in the co-culture setting. In contrast to protein expression, VPA at 2.5 mM had no impact on FL_NKG2D gene expression levels in γδ T cells (Figure 4A, upper panel). In PBMC both, FL_NKG2D and Tr_NKG2D transcript levels were increased after treatment with 2.5 mM VPA (Supplementary Figure 4). In γδ T cells or PBMC co-cultured with Panc89 and carefully removed from adherent tumor cells, FL_NKG2D and Tr_NKG2D transcripts were remarkably downregulated after treatment with 2.5 mM VPA. In PBMC co-cultured with PC-3, it was even further decreased (Supplementary Figure 4, upper panel) compared to VPA treatment, but unexpectedly this was not the case with γδ T cells (Figure 4A, upper panel). Thus, the gene expression level of NKG2D isoforms is not consistent in short-term expanded γδ T cell-lines and freshly isolated PBMC from such co-cultures with tumor cells.

Because our previous experiments showed a substantial release of the NKG2D ligands MICA, MICB, and ULBP-2, we also quantified the expression of these genes in the same co-culture set-up (Figure 4A, lower panel). In line with the increased protein levels, the gene expression of NKG2D ligands in both Panc89 and PC-3 was also increased upon treatment with 2.5 mM VPA. Of note, the level of expression was low in PBMC (Supplementary Figure 4) or γδ T cells co-cultured with untreated tumor cells, but was remarkably increased upon VPA treatment (Figure 4A). We applied a traditional *in silico* method, commonly used for graphical representation of microarray or sequencing experiments, to understand gene function and regulation. As a result of such clustering techniques, genes with similar expression patterns cluster together with similar cellular functions (24–29). Hence, we performed a statistical unsupervised hierarchical cluster analysis of NKG2D ligand and NKG2D receptor isoform genes under all experimental conditions (Figure 4B). Based on Euclidian distance method and average linkage rule, we found two distinct clusters, separating the full-length NKG2D receptor and ligands, representing functionally related genes. To our surprise, the truncated form of NKG2D receptor clustered together with ligands for NKG2D. As mentioned before, NKG2D ligand shedding and the truncated form of the NKG2D receptor are known to affect the expression and function of NKG2D receptor on effector cells. Thus, our results demonstrate a distinct association of NKG2D receptor transcript variants (truncated vs. full length), NKG2D ligand expression, and regulation upon HDAC inhibitor treatment.

### Flow Cytometric Analysis of H3K9 Acetylation in γδ T Cells

In addition to its effect on cell surface protein and intracellular cytokine expression (e.g., IL-4), VPA effectively targets class I HDAC proteins and induces H3K9 acetylation in human γδ T cells as previously shown by western blot analysis (17, 18). Considering the limitation in cell numbers and addressing the γδ T cell-tumor interaction by the use of co-culture setting in the present study, we addressed the combined effect of NKG2D ligand release during tumor cell co-culture and VPA-induced H3K9ac at the single cell level in γδ T cells using flow cytometry.

**Figure 5:**
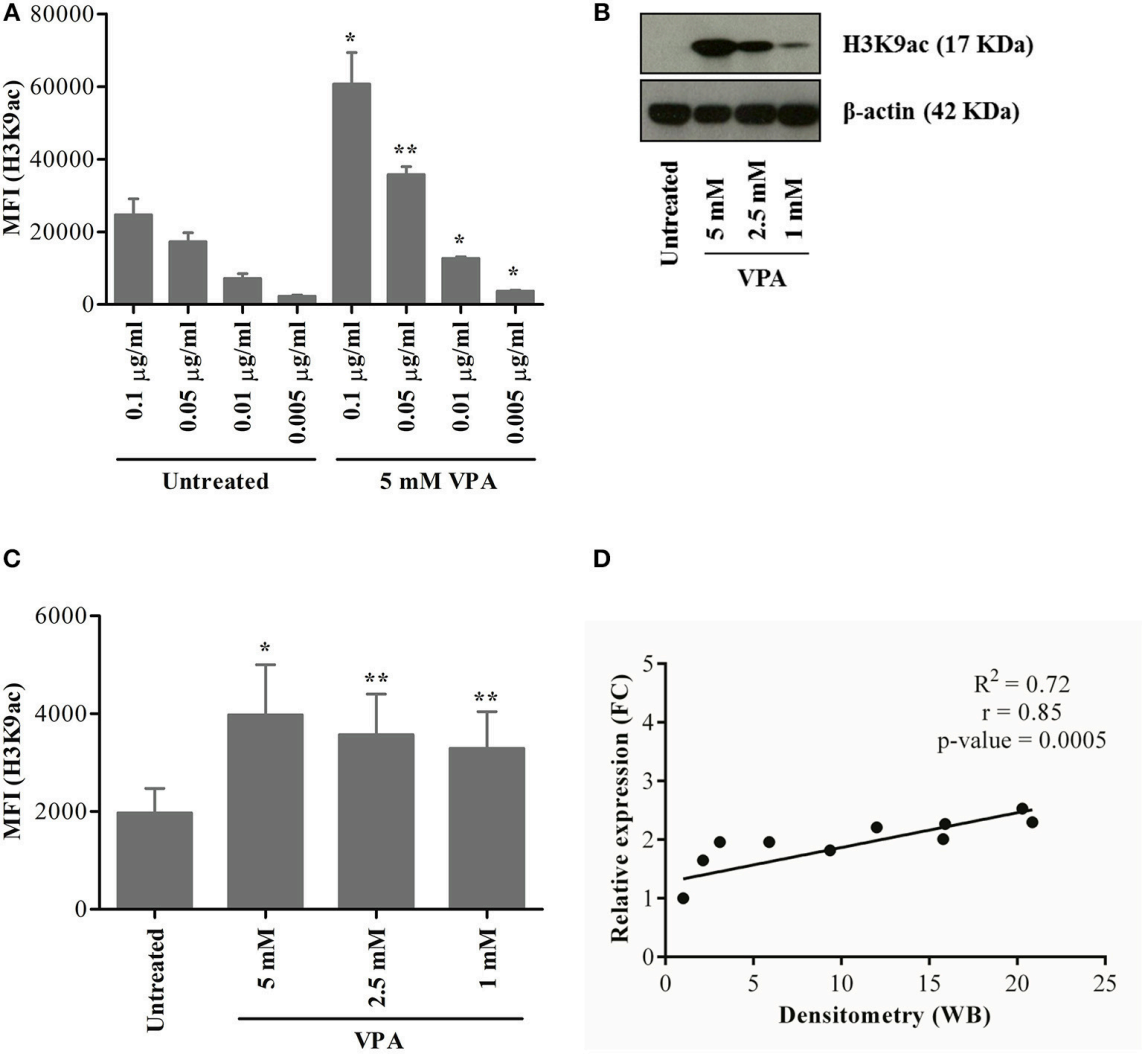
Establishment of a flow cytometry-based analysis of H3K9 acetylation. γδ T cells were derived from PBMC stimulated with zoledronic acid and IL-2 for 12 days. **(A)** γδ T cells treated or not with 5 mM VPA were stained with titrated concentration of H3K9ac antibodies. γδ T cells were also treated with 5, 2.5, and 1 mM VPA for 24 h and harvested for analysis by western blot **(B)** or flow cytometry **(C)**. For western blotting, β-actin was used as a loading control. For flow cytometry-based analysis, appropriate isotype control was used. **(D)** The ratio was first calculated between H3K9ac protein expression and loading control from western blot **(B)** and then the relative density was calculated by dividing the ratio of untreated condition by respective VPA treatment. Similarly, median fluorescence intensity (MFI) was first calculated by subtracting the MFI of isotype control from H3K9ac antibody **(C)** and then the relative expression was calculated by dividing MFI of untreated condition by respective VPA treatment. A correlation between relative density from western blot and relative expression from flow cytometry was calculated for statistical significance (*p*-value), correlation co-efficient (r), the coefficient of determination (R^2^) and represented using the scatter plot. Plots represented in the figure are based on values from three to five independent experiments. *P*-values of significance for <0.05 and 0.01 are indicated by ^*^ and ^**^, respectively.

To this end, we adapted a previously reported method to investigate changes in protein expression associated with H3K9ac in γδ T cells based on the staining with a monoclonal anti-H3K9ac antibody (30, 31). The highest concentration (0.1 μg/ml) of Pacific Blue-labeled H3K9ac antibody gave a higher background in untreated γδ T cells, which decreased substantially with decreasing antibody concentrations (Figure 5A, left; Supplementary Figure 5A). As shown by western blot detecting a single distinct band at 17 Kda, VPA-induced H3K9ac in a dose-dependent manner in γδ T cells (Figure 5B). For flow cytometry, H3K9ac antibody at 0.01 μg/ml concentration was chosen further based on the ratio of median fluorescence intensity (MFI) between untreated and 5 mM VPA-treated γδ T cells (Figure 5A, right). Our results with VPA-dose-dependent decrease in H3K9ac protein determined by flow cytometry (Figure 5C; Supplementary Figure 5B) further substantiated the use of 0.01 μg/ml H3K9ac antibody since such VPA-dose-dependent decrease was not observed with 0.005 μg/ml (data not shown). Overall, H3K9ac analysis by flow cytometry strongly correlated (*R*^2^ = 0.72, *r* = 0.85, *p*-value = 0.0005) with the densitometric analysis of conventional western blot (Figure 5D), but was highly sensitive toward the co-staining with other antibody-conjugates in the flow cytometry.

To further validate the flow cytometry-based epigenetic analysis, we performed control experiments to verify the specificity of the H3K9ac antibody. ImageStream cytometry was used to analyze the co-localization of H3K9ac with DRAQ5™ (a DNA intercalating dye, which stains living cells only) in human γδ T cells treated or not with 5 mM VPA. Complete co-localization of H3K9ac within the nuclear stain confirms “nuclear specificity” of the H3K9ac antibody. Treatment with 5 mM VPA enhanced H3K9ac staining (Supplementary Figure 5). Moreover, the co-localization of H3K9ac and DNA was strikingly enhanced only after treatment with VPA (Supplementary Figure 5C). The weak expression of H3K9ac observed in untreated γδ T cells (Supplementary Figure 5D) corresponds to the low constitutive expression of H3K9ac in the cell and is referred to as “H3K9ac^low^” (Figure 5; Supplementary Figure 5C). This can be clearly distinguished from 5 mM VPA-treated γδ T cells showing strong inducible expression of H3K9ac, referred to as “H3K9ac^high^” (Supplementary Figure 5E). Taken together, the single cell epigenetic analysis revealed specificity for nuclear H3K9 acetylation distinguishing levels of H3K9ac expression in γδ T cells, thus further corroborating previous single cell epigenetic analysis of CD8 T cells (31).

### VPA Affects the Distribution of H3K9ac-Associated Memory γδ T Cells Under *in vitro* Tumor Co-culture Conditions

**Figure 6:**
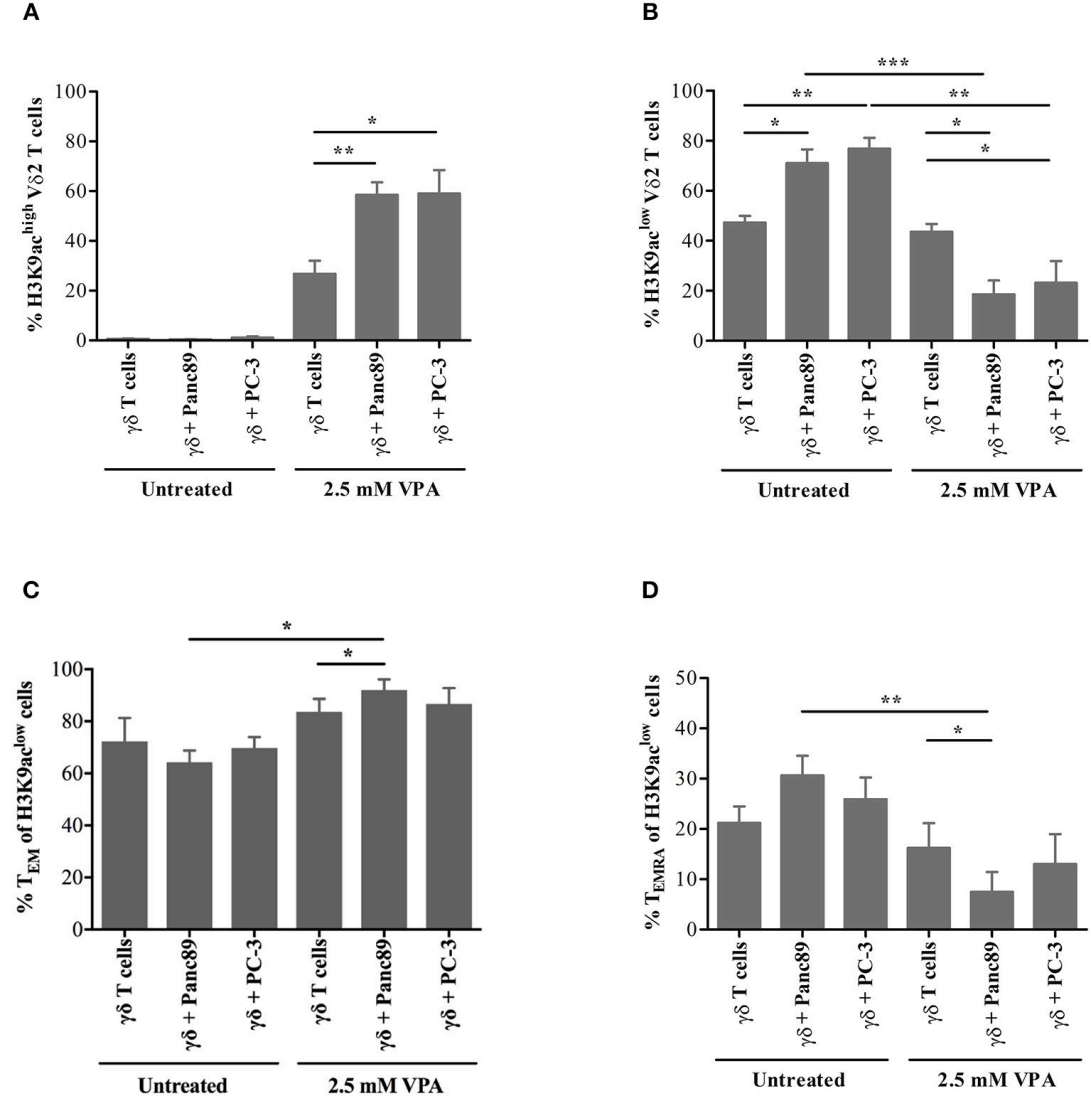
H3K9 acetylation-associated memory phenotype of γδ T cells upon *in vitro* co-culture with tumor cells. γδ T cell lines generated from 12 days zoledronic acid-stimulated PBMC were co-cultured with 2.5 mM VPA pre-treated Panc89 and PC-3 cells. After 24 h of co-culture, cells were harvested and stained with FITC-conjugated Vδ2, APC-H7-conjugated CD27, PE-Cy7-conjugated CD45RA and Pacific Blue-conjugated H3K9ac antibodies. Based on flow cytometry gating strategy described in the Supplementary Figure 5, Vδ2-gated cells were defined as **(A)** H3K9ac^high^ and **(B)** H3K9ac^low^ in solo- or co-culture with Panc89 and PC3 cells in the presence and absence of 2.5 mM VPA. The proportions of H3K9ac^low^ cells within Vδ2 T cells with **(C)** T_EM_ and **(D)** T_EMRA_ phenotype from the co-culture with Panc89 or PC-3 with or without 2.5 mM VPA treatment are shown. Data represented as mean ± S.E. of four independent experiments. Statistical significance is shown by ^*^, ^**^, ^***^ for *p*-values < 0.05, 0.01 and 0.005, respectively.

Having established the ultra-sensitive, flow cytometry-based method of H3K9ac analysis in γδ T cells, we further extended this approach to evaluate the distribution of γδ memory T-cell phenotypes. To this end, we used a similar experimental set-up as applied to address NKG2D receptor expression. γδ T cells were harvested from co-cultures with Panc89 and PC-3 tumor cells or from solo-cultures treated or not with 2.5 mM VPA. Consistent with the previous observations, we detected H3K9ac^low^ and H3K9ac^high^ expression in γδ T cells upon 2.5 mM VPA treatment, irrespective of tumor co-culture or solo-culture (flow cytometry gating strategy illustrated in the Supplementary Figure 6). The proportion of H3K9ac^high^ cells among Vδ2 T cells was significantly increased in the co-culture with Panc89 and PC-3 cells after 2.5 mM VPA treatment (Figure 6A), while the percentage of H3K9ac^low^ Vδ2 T cells was significantly reduced (Figure 6B). This clearly reflects the change in H3K9ac levels as an effect of VPA treatment upon co-culture with tumor cells. Interestingly, the constitutive expression of H3K9ac in γδ T cells was significantly increased in Panc89 and PC-3 co-cultures (Figure 6B). Thus, the *in vitro* co-culture with Panc89 and PC-3 tumors modulates epigenome-wide inducible histone acetylation levels in γδ T cells, which might have consequences for the functional cellular response. As a first step to address this, we analyzed memory subset phenotypes based on the surface markers CD27 and CD45RA (as described by Dieli et al. (32)) within H3K9ac^low^ and H3K9ac^high^ Vδ2 T-cell populations. Memory phenotype distribution of Vδ2 T cells associated with H3K9ac^high^ remained unaffected and also the distribution of naïve (T_N_) and central memory (T_CM_) phenotypes of H3K9ac^low^ Vδ2 T cells did not change remarkably (data not shown). However, a substantial change in the relative distribution of effector memory (T_EM_) cells (defined as CD27^−^CD45RA^−^) and terminally differentiated (T_EMRA_) cells (defined as CD27^−^CD45RA^+^) was observed (Figures 6C,D). Of note, the significant increase in the proportion of TEM within H3K9ac^low^ Vδ2 T cells (Figure 6C) and the decrease in the proportion of TEMRA within H3K9ac^low^ Vδ2 T cells (Figure 6D) were found only upon co-culture with Panc89 tumor cells treated with 2.5 mM VPA, and not in the case of PC-3 tumor cells. Thus, taking all results together, it appears that Vδ2 T cells constitutively expressing low levels of H3K9ac might revert to the T_EM_ phenotype under the *in vitro* microenvironment of pancreatic carcinoma in response to inhibition of histone deacetylase enzymes.

## Discussion

Previous reports from our group and others have shown that the differential mechanisms of NKG2D ligand release are related to the origin of tumor cells and the allelic polymorphisms of MICA (5, 6). In this study, we used epigenetic inhibitors for further analysis of NKG2D ligand shedding. When we targeted proteins involved in histone and DNA modifications, we found that only inhibitors of HDACs (from the family of epigenetic “erasers”) modulated NKG2D ligand expression and/or release. Heterogeneity in the release of NKG2D ligands based on the tumor origin was also observed after treatment with the HDAC inhibitor VPA. Interestingly, PC-3 cells released MICA directly into the culture supernatant after VPA treatment at the concentrations tested, suggesting that PC-3 cells are not exclusively dependent on exosome release. This substantial change in the mechanism of MICA release might be due to the MICA heterozygosity of PC-3 cells, exhibiting MICA^*^008:01:01 (A5.1) and MICA^*^012:01 (A4) allelic polymorphism (6). However, previous reports have not described such a switch in the release of MICA after treatment with VPA and its possible association with heterozygous MICA expression (33, 34). In human ovarian and cervical cancer cell lines, Trichostatin A (TSA) and sodium butyrate had a similar effect on MICA/B induction, while VPA was less effective (33). But as in human hepatoma cells (34), the effect of VPA was consistent with what we observed in our study using pancreatic and prostate cancer cells. Chitadze et al. demonstrated that shedding of MICA/B from Panc89 cells was mediated by metalloproteases (6). But, at this point, it is not clear whether VPA-induced NKG2D ligand release observed in the present study is due to ADAM-10/17- or MMP-mediated proteolysis. In addition to the heterozygosity of MICA and the possible mechanism of proteolytic cleavage, the origin of tumor cells might also play an important role in the response to VPA.

VPA has been used *in vitro* at concentrations ranging from 0.5 to 10 mM (35–37). To study the modulation of MICA expression and release from tumor cells, Armeanu et al. used 1 mM VPA for human hepatoma cells. Their results showed a differential induction of NKG2D ligands by VPA on malignant and non-malignant cells (34). Yamanegi et al. also used VPA at 0.5- and 1-mM concentration for human osteosarcoma cells revealing increase in the cell-surface but not soluble form of MICA/B (38). In contrast, our study revealed only minor changes in the shedding of MICA from Panc89 cells and no changes at all in PC-3 cells after treatment with 1 mM VPA. In fact, NKG2D ligand (MICA/B and ULBP-2) gene expression was remarkably induced with 2.5 mM VPA both on malignant (pancreatic carcinoma cells Panc89 and prostate carcinoma cells PC-3) and non-malignant cells (PBMC and γδ T cells). The use of 2.5 mM VPA concentration to induce MICA protein expression is consistent with a previous study performed with Hodgkin lymphoma cells (39). The ULBP-1 expression is known to be associated with proteasome regulation (40). Remarkably, VPA failed to induce ULBP-1 expression on Panc89 and PC-3 cells. Nevertheless, the treatment of melanoma cells with 1 mM VPA has been shown to induce only MICA, MICB and ULBP-2, mediated by the ERK pathway (41), which is also consistent with our results using Panc89 and PC-3 tumor cells. The role of specific signaling pathways like ERK1/2 and AKT in cellular responses of Panc89 and PC-3 to VPA treatment remains to be investigated.

Another important aspect of our study is the direct evidence linking functional cellular responses of γδ T cells to epigenetic mechanisms. In this regard, we first showed down-regulation of the NKG2D receptor on γδ T cells at the protein and gene expression level upon VPA treatment. It has been noted that NKG2D ligand shedding down-modulates NKG2D receptor expression on effector cells, such as NK cells (8). In contrast, however, Deng and coworkers showed increased NKG2D receptor expression (9). At the protein level, we observed down-regulation of the NKG2D receptor on γδ T cells in tumor cell co-cultures, but the expression was maintained on lymphocyte-gated freshly isolated PBMC (including NK cells), indicative of a primarily γδ T-cell-specific response. Down-regulation of the full-length form of the NKG2D receptor and of NKG2D ligands was corroborated at the level of gene expression. The truncated NKG2D receptor plays an important role in down-regulating the expression of the full-length NKG2D receptor (23), which is also a characteristic feature of NKG2D ligand-mediated shedding. Of note, this functional similarity is further strongly supported by the cluster analysis, which grouped the truncated form of the NKG2D receptor together with all NKG2D ligands (MICA/B and ULBP2), though a slight increase in truncated NKG2D receptor in γδ T cells due to VPA treatment was observed. But such increase was not found in co-culture with Panc89 upon VPA treatment. This observation supports the view that in addition to other possible mechanisms, the truncated NKG2D form may be involved in the modulation of VPA-induced NKG2D receptor expression in the co-culture with γδ T cells. Additionally, a decrease in the gene expression of NKG2D receptor (both full-length and truncated form) after VPA treatment was observed during the interaction with Panc89, but not with PC-3. The differences in the mechanisms of NKG2D ligand release are likely affected by VPA-induced epigenetic modification or *vice versa*. This, however, needs to be studied further in detail. But it is clear from our current and previous other studies that the protein/gene expression of NKG2D ligands is heterogenous, cell type-dependent and most likely regulated at the prost-transcriptional level (6, 19, 20, 42, 43).

Furthermore, we provided evidence for a direct link between γδ T-cell memory subset distribution and epigenome-wide H3K9ac (an activating marker) expression. Although the distribution of memory subsets was not altered within the γδ T-cell population with inducible expression of H3K9ac (i.e., H3K9ac^high^), we are the first to show that the memory phenotypes of γδ T cells are most likely modulated through the constitutive H3K9ac expression (i.e., within the H3K9ac^low^ subset). We previously reported that H3K9ac expression induced by VPA (which most likely corresponds to the H3K9ac^high^ population defined in this study) leads to the expression of a non-secreted isoform of IL-4 and the regulation of c-Jun transcription factor in γδ T cells, but not in αβ T cells (18). Thus, this is a very important finding with respect to the effect of the HDAC inhibitor under these *in vitro* conditions mimicking the tumor microenvironment, specifically in the case of pancreatic ductal adenocarcinoma (PDAC). The reversion of the memory phenotype distribution from T_EMRA_ to T_EM_ in γδ T cells supports the view that the γδ T_EM_ subset is mainly associated with the secretion of pro-inflammatory cytokines, cytotoxicity and the expression of homing receptors for inflamed tissues (32, 44, 45). It has already been described that γδ T cells have the capacity to elicit anti-tumor response mediated through the perforin-granzyme pathway, not solely depending on NKG2D receptor-ligand signaling, by the T_EM_ phenotypic subset (45, 46). This seems to hold true based on the analysis of lysosomal degranulation using the marker CD107a in our study. In the present experimental model, γδ T cells exhibited degranulation in response to Panc89 (pancreatic adenocarcinoma cells), but not to PC-3 (prostate carcinoma cells), which corroborates the functional role of γδ T_EM_ subset. In the case of oral-, colon-carcinoma cells and B lymphoblastic cell line (47) and osteosarcoma (48), γδ T cells exerted this cytotoxicity mainly via TCRγδ- and Fas-/perforin-mediated mechanisms, or by blocking PD-1 signal after VPA and Zol treatment (with a minor role of NKG2D and TRAIL), respectively. In the future, we will analyze the expression of perforin and granzymes, PD-1 and NKG2D to explore the mediators of cytotoxicity, exerted by T_EM_ γδ T cells under such *in vitro* conditions mimicking the tumor microenvironment.

The results presented here on the effects of HDACi VPA may have implications to further improve γδ T-cell-based immunotherapy (49, 50). A clinical phase I study with multiple myeloma patients has already proved its safety and emerged as a promising approach for the adoptive transfer of zoledronic acid-activated Vγ9Vδ2 T cells (51). This study specifically revealed the significant increase in the CD27^−^CD45RA^+^ T_EM_ subpopulation within Vγ9Vδ2 T cells. A combined approach with epigenetic modifiers, such as VPA might further increase the efficacy but this needs to be tested in preclinical studies using suitable *in vivo* models. The combination of γδ T cell based immunotherapy with VPA treatment might be a particularly promising translational strategy for the treatment of patients with pancreatic ductal adenocarcinoma (PDAC).

## Materials and Methods

### Tumor Cell Culture and Reagents

Panc89, pancreatic ductal adenocarcinoma has been previously characterized (52) and PC-3, prostate cancer cell line was obtained from ATCC. Both tumor cell lines and effector cells were cultured in RPMI-1640 medium (Gibco^Ⓡ^) supplemented with 10% heat-inactivated Fetal Calf Serum (FCS; Gibco), 1% Penicillin/Streptomycin (PS; Biochrom), hereafter referred to as growth medium. Tumor cell lines were maintained in 175 cm^2^ Cellstar^Ⓡ^ cell culture flasks (Greiner Bio-One). All cell cultures were kept at 37 °C in a humidified atmosphere with 5% CO^2^. Valproic acid (VPA; #P4543), Trichostatin A (TSA; #T8552), 4-Phenylbutyric acid (4-PBA; #P21005), Epigallocatechin gallate (EGCG; #1236700), 5-Aza-2′-deoxycytidine (Decitabine; #A3656), Curcumin (#C1386) were used at indicated concentrations; all reagents were purchased from Sigma-Aldrich.

### Effector Cell Cultures

PBMC were isolated from healthy donors (Department of Transfusion Medicine, UKSH, Kiel, Germany) with approval by the local Ethics Committee (D405/10). PBMC were either used freshly or stimulated with 2.5 μM zoledronic acid (kindly provided by Novartis) in the presence of 50 IU/ml IL-2 (Novartis). Furthermore, IL-2 was added every other day until day 12. Day 12 cultures routinely contained >90% Vγ9Vδ2-expressing γδ T cells.

### Analysis of NKG2D Ligand Expression on Tumor Cells by Flow Cytometry

Epigenetic inhibitors VPA, TSA, 4-PBA, EGCG, Decitabine or Curcumin were used at indicated concentrations to study the modulation of NKG2D ligand expression and release from tumor cells. 0.8 × 10^6^ tumor cells in growth medium were seeded in 12-well plates, either treated with respective epigenetic inhibitors, DMSO as solvent control for TSA, Decitabine and Curcumin treatment or left untreated. Cells were harvested, washed and surface stained with the following anti-human antibodies: PE-conjugated anti-human MICA (clone 159227; #FAB1300P), Alexa Fluor 700-conjugated anti-human MICB (clone 236511, #FAB1599N), PerCp-conjugated anti-human ULBP-1 (clone 170818, #FAB1380C) and APC-conjugated anti-human ULBP2/5/6 (clone 165903, #FAB1298A) together with respective isotype controls (all from R&D Systems). After this cell surface staining, cells were immediately measured on a LSR Fortessa flow cytometer (BD Biosciences). Data was analyzed using the FlowJo software (FlowJo LLC). Median fluorescence intensity was calculated by subtracting the median intensity of the isotype control antibody from the respective test antibody fluorescence.

### NKG2D Ligand Shedding (ELISA)

Culture supernatants were spun down to remove debris. Flat-bottom 96-well Maxisorp Nunc Immunoplates (Thermo Fisher Scientific) were used for coating with antibodies to quantitate soluble MICA, MICB, ULBP-1, ULBP-2 (#DY1300, #DY1599, #DY1380, #DY1298, respectively; all from R&D Systems) by ELISA according to manufacturer's protocol.

### Co-culture Assays

For co-culture assays, 0.8 × 10^6^ tumor cells per ml of growth medium were treated with 2.5 mM VPA for 24 h in 12 well cell culture plates (Greiner Bio-One). Afterwards, 1 × 10^6^ effector cells (PBMC or γδ T cells) were added to the culture for another 24 h without changing medium. Thereafter, non-adherent cells (i.e., PBMC or γδ T cells) were carefully resuspended and harvested for cytotoxicity, gene expression and flow cytometric analysis.

### Flow Cytometric Analysis of Effector Cells

As described above, effector cells (i.e., γδ T cells or PBMC) were harvested after 24 h of co-culture with Panc89 and PC-3 cells. Cells were washed once with cold Dulbecco's PBS (Cell Concepts) and surface stained with the following mAb: FITC anti-Vδ2 (clone IMMU389, #IM1464, Beckman Coulter), PE anti-NKG2D (clone 149810, #FAB139P, R&D Systems), APC-Cy7 anti-CD27 (clone M-T271, #560222, BD Horizon), PE-Cy7 anti-CD45RA (clone L48, #337167, BD Biosciences). The effector cells were stained with respective antibodies for the NKG2D receptor and ligands by gating on Vδ2 T cells or on lymphocytes (based on forward-side scatter properties) of PBMC co-culture. Afterwards, cells were either measured immediately or otherwise processed further for intranuclear staining. For intranuclear staining, surface stained cells were permeabilized and fixed using BD Pharmingen Transcription Factor Buffer set (#562574, BD Biosciences). Staining was performed using Pacific Blue-conjugated anti-human H3K9ac rabbit monoclonal antibody (clone C5B11, #11857S) or Pacific Blue-conjugated anti-rabbit isotype control monoclonal antibody (clone DA1E, #9078S; both from Cell Signaling Technology). 10,000 Vδ2 T cells or lymphocyte-gated PBMC were acquired on a LSR Fortessa flow cytometer (BD Biosciences). FACSDiva software was used for acquisition, while data analysis was done using FlowJo software (FlowJo LLC).

### Degranulation Assay

As described in the methods section Co-culture Assays and in the Supplementary Figure 1, γδ T cells were co-cultured with Panc89 or PC-3 cells with or without 2.5 mM VPA and/or 2.5 μM Zoledronate (Zol). During the last 4 h of solo-/co-culture, 50 μl PE anti-CD107a mAb (50 ng/ml, clone: H4A3, #555801, BD Biosciences) was added directly, and after 1 h i.e., during the last 3 h, monensin (3 μM, EMD Millipore/Calbiochem) was added. γδ T cells were harvested from the solo-/co-cultures and were stained for FITC anti-Vδ2 mAb. 10,000 Vδ2 T cells were acquired on a LSR Fortessa flow cytometer (BD Biosciences) and FlowJo software (FlowJo LLC) was used for the data analysis.

### Gene Expression Analysis of NKG2D Receptor and Ligands

Human PBMC, γδ T cells, Panc89, and PC-3 cells were harvested from co-culture experiments as described above. Effector or target tumor cells were washed once with cold Dulbecco's PBS, resuspended in peqGOLD TriFast solution (#30-2010, Peqlab) and stored at −80 °C until further use. RNA was extracted and transcribed into cDNA using the cDNA synthesis kit (#8994-A30, AmpTec). For PCR amplification, respective PCR primers for NKG2D receptor and ligands were used at the annealing temperature 60 °C (details in Table 1). qPCR data were analyzed using relative quantitation method by normalizing with the mean Ct value of the housekeeping genes (β2-microglobulin, β-actin, and 18S RNA). The calculated relative expression values of NKG2D receptor and ligand genes were imported and visualized as heatmap based clustering using CIMminer (https://discover.nci.nih.gov/cimminer/home.do). The Euclidian distance method with average linkage rule was used (28, 29).

**Table 1:** PCR primers used in this study.

**Gene name**	**Primer**	**Sequence**
**NKG2D RECEPTOR**		
FL_NKG2D	Forward	GCTGTATTCCTAAACTCATTATTCAACC
	Reverse	CTGCCAAGATCCATTTGTTG
Tr_NKG2D	Forward	TTCTGCTGCTTCATCGCTGT
	Reverse	TGGACTAATAGCAAAAATGTGACAA
Both_NKG2D	Forward	CCTCTCTGCGGTAGACGTG
	Reverse	GACATCTTTGCTTTTGCCATC
**NKG2D LIGANDS**		
MICA	Forward	AGGGTCTGTGAGATCCATGAAGAC
	Reverse	CCTGACGTTCATGGCCAAGG
MICB	Forward	ACCTTGGCTATGAACGTCACA
	Reverse	CCCTCTGAGACCTCGC
ULBP-2	Forward	GCAAGGATGTCTTGTGAGCA
	Reverse	GGCCACAACCTTGTCATTCT
**HOUSEKEEPING GENES**		
β2-microglobulin	Forward	GGGTTTCATCCATCCATCCGACA
	Reverse	ACACGGCAGGCATACTCATC
β-actin	Forward	CTGAACCCCAAGGCCAAC
	Reverse	CAGAGGCGTACAGGGATAGC
18S RNA	Forward	GACTCAACACGGGAAACCTC
	Reverse	AGACAAATCGCTCCACCAAC

*To analyze the mRNA expression of NKG2D receptor and ligand following PCR primers were used. Housekeeping genes were used as internal control. PCR primers were designed using the Web-based primer3 software (http://primer3.wi.mit.edu/) and purchased from TIB MOLBIOL*.

### Western Blot

Total H3K9ac in human γδ T cells was analyzed by western blot as described previously (17, 18). Briefly, 1 × 10^6^ per ml of γδ T cells were either treated with 5 mM, 2.5 mM, 1 mM VPA or left untreated. After 24 h of treatment, cells were harvested and lysed. 10 μg of protein was separated by SDS-PAGE, transferred to nitrocellulose and analyzed with a primary rabbit monoclonal antibody against H3K9ac (clone C5B11, #9649, Cell Signaling Technology) and β-actin (clone AC-15, #A5441, Sigma-Aldrich). HRP-conjugated secondary anti-rabbit antibodies (#NA9340V, GE Healthcare) were used for detection and visualized by the enhanced chemiluminescence system (#RPN2106, GE Healthcare).

### ImageStream Analysis

The ImageStream analysis was performed to study co-localization between H3K9ac and nuclear staining as published previously (18). Shortly, 1 × 10^6^ per ml of γδ T cells were treated with 5 mM VPA or left untreated as a control. After 24 h of treatment, γδ T cells were harvested and stained with Pacific Blue-labeled anti-human H3K9ac antibody. At the end of intracellular H3K9ac staining, 1 μM DRAQ5™ (#ab108410, Abcam plc) was added to the cell suspension. After incubation for 15 min at 37 °C, cells were washed once with cold PBS. Unstained or stained γδ T cells with or without 5 mM VPA treatment were acquired immediately on an ImageStreamX Mark II imaging flow cytometer (Merck Millipore). IDEAS software (v6.0, Amnis) was used to acquire images (60×) and statistical calculations. Special co-localization wizard of the IDEAS software was applied for single cell analysis.

### Statistical Analysis

Statistical analysis was performed using PrismGraph software. Statistical significance was calculated using Student's *t*-test to evaluate the significant differences between two experimental conditions (53). *p*-Value <0.05 was considered significant and were displayed as ^*^ for <0.05, ^**^ for <0.01 and ^***^ for <0.001.

## Author Contributions

JB and DK conceptualized and designed the experiments. JB, SD, and AD developed methodology. JB, ESQ, JF, CMD, GC, and ML acquired the data. JB, ESQ, JF, CMD, AS, GC, and ML analyzed and interpreted the data. JB, CMD, AS, GC, ML, and DK wrote the manuscript. ESQ, JF, and ML provided administrative and technical help. DK supervised the study.

### Conflict of Interest Statement

The authors declare that the research was conducted in the absence of any commercial or financial relationships that could be construed as a potential conflict of interest.
