# Supplementary material for: Histone Deacetylase Inhibitor Modulates NKG2D Receptor Expression and Memory Phenotype of Human Gamma/Delta T Cells Upon Interaction With Tumor Cells
Source: Front Immunol. 2019 Mar 27;10:569. doi: 10.3389/fimmu.2019.00569 (PMC6445873; doi:10.3389/fimmu.2019.00569)
Supplement: Supplementary file 1 [file Table_1.docx]

Supplementary Material

Histone deacetylase inhibitor modulates NKG2D receptor expression and memory phenotype of human gamma/delta T cells upon interaction with tumor cells

Jaydeep Bhat ^1^, Samuel Dubin^1^, Alexandra Dananberg^1^, Elgar Susanne Quabius^1,2^, Juergen Fritsch^1^, C. Marie Dowds^1,3^, Ankit Saxena^4^, Guranda Chitadze^1^, Marcus Lettau^1^, Dieter Kabelitz^1*^

^1^ Institute of Immunology, University Hospital Schleswig-Holstein (UKSH) Campus Kiel, D-24105 Kiel, Germany

^2^ Dept. of Oto-Rhino-Laryngology, UKSH Campus Kiel, D-24105 Kiel, Germany

^3^ Institute of Clinical Molecular Biology, Kiel University, D-24105 Kiel

^4^ National Heart, Lung and Blood Institute, National Institutes of Health, Bethesda, MD, United States

*** Correspondence:**Dieter Kabelitz
[dietrich.kabelitz@uksh.de](mailto:dietrich.kabelitz@uksh.de)

# Supplementary Figures

**Supplementary Figure 1:** **Experimental strategy to analyze the modulation of NKG2D receptor - ligand mediated functional responses to epigenetic inhibitors.** NKG2D ligand (MICA, MICB, ULBP-1 and ULBP-2/5/6) expression and release from the pancreatic carcinoma cell line (Panc89) and prostate carcinoma cell line (PC-3) was measured after 24 hrs treatment with epigenetic modifiers. (A) After 24 hrs, tumor cells were analyzed for NKG2D ligand expression and culture supernatants were collected to quantitate NKG2D ligands released from the same experiment. (B) Otherwise, effector cells (freshly isolated PBMC or γδ T-cell lines) were added to tumor cells for co-culture at a 1:1 ratio. After 24 hrs of co-culture, effector and/or tumor cells were harvested and analyzed for NKG2D receptor expression and combined H3K9ac - T-cell memory marker expression by flow cytometry, or NKG2D receptor - ligand gene expression by qPCR.

**Supplementary Figure 2: Representative histograms for NKG2D ligand staining on tumor cells.** As schematically shown in the Supplementary Figure 1, Panc89 and PC-3 cells were treated with varying concentration of inhibitors for HDAC, HAT and DNMT for 24 hrs. Afterwards, cells were harvested and stained with PE anti-MICA, Alexa Fluor 700 anti-MICB, PerCp anti-ULBP-1, APC anti-ULBP-2 or with their respective isotype control antibodies. Histograms represent one out of three independent experiment performed for (A) Panc89 and (B) PC-3 either untreated or treated with VPA at the concentrations of 5-, 2.5- and 1.25- mM. The dark gray color histogram shows staining for the antibody of NKG2DLs, while the light gray color histogram above dark color shows staining for the respective isotype control antibody. The flow data was analyzed using FlowJo software.

**Supplementary Figure 3: Histone deacetylase inhibitor does not modulate NKG2D ligand cell surface expression and release from γδ T cells except ULBP-2.** In a similar set-up of experiments as used for tumor cells (Panc89 and PC-3), γδ T cells were treated with 5-, 2.5- and 1.25- mM VPA for 24 hrs. Afterwards, cells were harvested and analyzed for (A) MICA, (B) MICB, (C) ULBP-1 and (D) ULBP-2 cell surface expression by flow cytometry. From the same experiments, culture supernatants were also harvested and analyzed for soluble form of (E) MICA, (F) MICB, (G) ULBP-1 and (H) ULBP-2 using respective ELISA kits. The median fluorescence intensities (MFI) were calculated by subtracting MFI of isotype control antibody from the respective NKG2D ligand antibody. The mean ± S.E. of median fluorescence intensities obtained from three independent experiments is represented here.

**Supplementary Figure 4: Histone deacetylase inhibitor-mediated regulation of NKG2D receptor and ligand gene expression.** In a similar set-up of experiments as in Figure 4, PBMC were treated with 2.5 mM VPA or left untreated and co-cultured with and without Panc89 and PC-3. After 24 hrs, PBMC from co-culture, or tumor cells from solo-culture (as an internal control) were harvested and analyzed for mRNA expression of full length NKG2D (FL_NKG2D), truncated NKG2D (Tr_NKG2D), MICA, MICB and ULBP-2/5/6. mRNA levels were calculated as relative expression values compared to the mean Ct value of the housekeeping genes (β-actin, β2-microglobulin and 18S). Graphs represented are mean values ± S.E. of 3 independent experiments with p-values <0.001, 0.05, 0.01 as ***, **, *.

**Supplementary Figure 5: ImageStream analysis showing binding specificity of the histone acetylation-specific antibody.** γδ T cells were treated or not for 24 hrs with 5 mM VPA. Untreated and 5 mM VPA-treated γδ T cells were permeabilized and stained with Pacific Blue-conjugated H3K9ac antibody for flow cytometric analysis and additionally with 1 µM DRAQ^TM^ (a nuclear dye, staining live cells) for ImageStream analysis using co-localization wizard. (A-B) are the representative histograms for the results summarized in figure 5A and C, respectively. The dark gray color histogram shows staining for Pacific Blue anti-H3K9ac, while light gray color histogram above it shows corresponding isotype control staining. The data was analyzed using FlowJo software. (C) Frequency plot represents change in fluorescence signal intensities for H3K9ac staining with three distinct fractions, ‘NS’ for not stained, ‘low’ for untreated Vδ2 T cells represented using the red line and ‘high’ for VPA-treated Vδ2 T cells represented using the green line. (D) Untreated γδ T cells with minimal background staining for H3K9ac with living cells and (E) 5 mM VPA-treated γδ T cells clearly showing enhanced nuclear H3K9ac staining. 5000 cells were acquired in three independent experiments and three representative cells are shown in the image.

**Supplementary Figure 6: Strategy used for flow cytometric analysis of combined histone acetylation and memory γδ T-cell markers.** As described in Figure 6, human γδ T cells were collected from tumor cell co-cultures and analyzed for memory markers in combination with H3K9ac. For the flow cytometry data analysis, cells were first gated based on FSC vs SSC as lymphocytes and SSC vs Vδ2-FITC was used to distinguish Vδ2 T cells from non-Vδ2 T cells. These Vδ2 T cells were further defined as H3K9ac^low^ and H3K9ac^high^ using H3K9ac-pacific blue staining intensity in the histogram. Based on these H3K9ac expression levels by setting gate as H3K9ac low and high, cells were analyzed for CD27-APC-H7 and CD45RA-PE-Cy7 within these gates. The blue color population in the dot plot represents respective isotype control antibody staining, while the red color population is for CD27-APC-H7 and CD45RA-PE-Cy7. The representative analysis is from γδ T cell solo-culture of the same experiment.
