# Supplementary figures and images for: Histone Deacetylase Inhibitor Modulates NKG2D Receptor Expression and Memory Phenotype of Human Gamma/Delta T Cells Upon Interaction With Tumor Cells
Source: Front Immunol. 2019 Mar 27;10:569. doi: 10.3389/fimmu.2019.00569 (PMC6445873; doi:10.3389/fimmu.2019.00569)

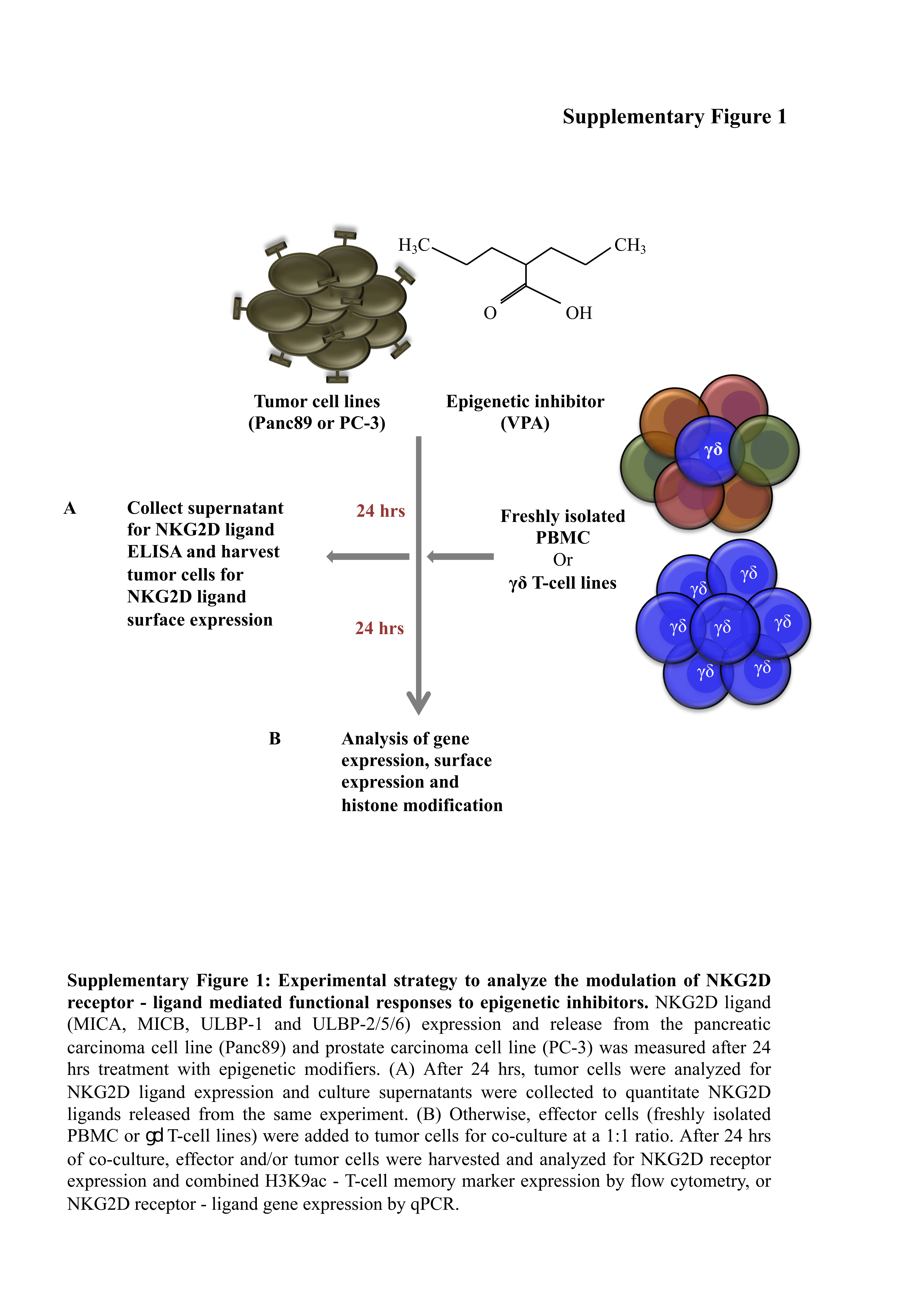

Supplement: Supplementary file 2 [file Image_1.TIFF]

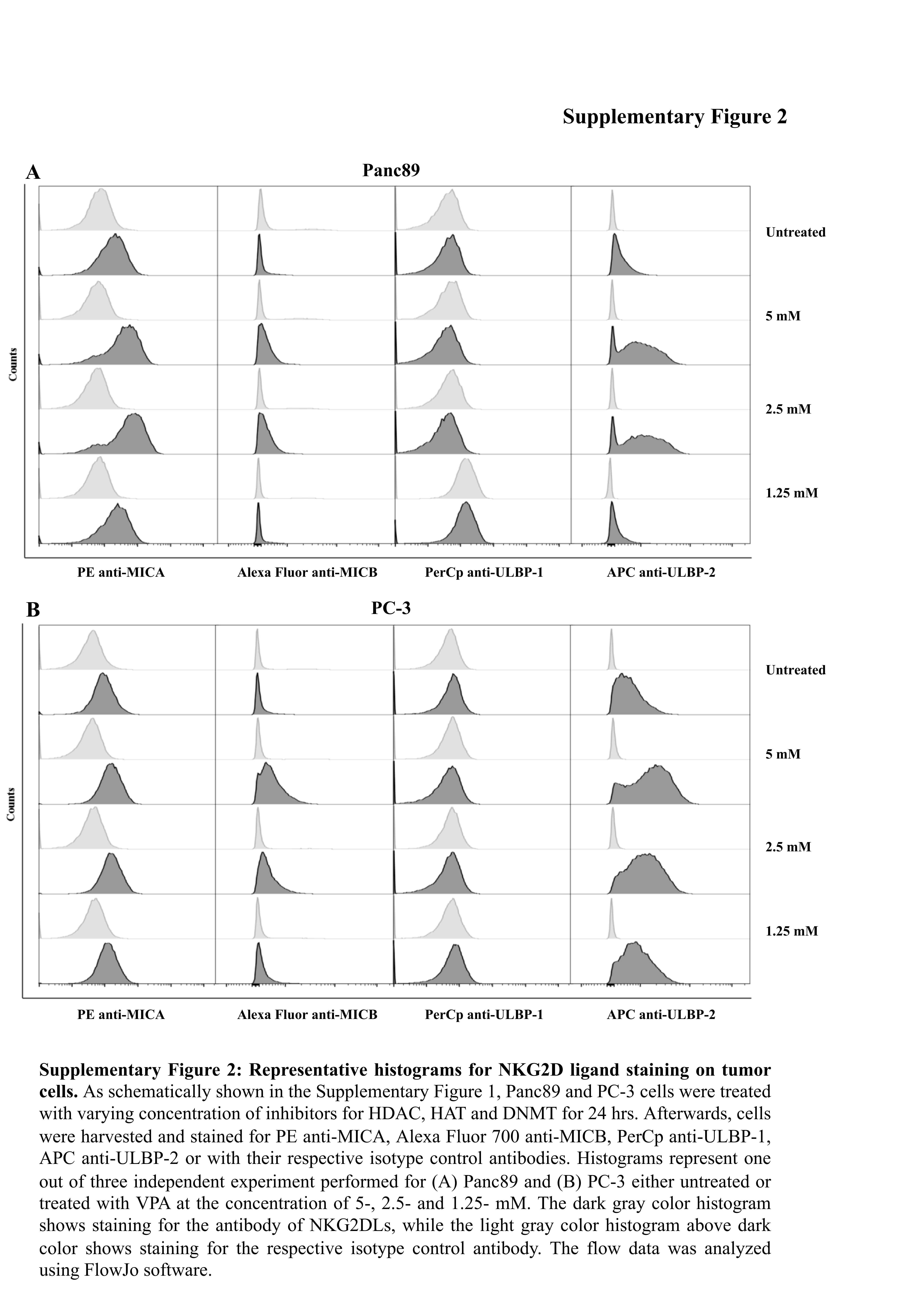

Supplement: Supplementary file 3 [file Image_2.TIFF]

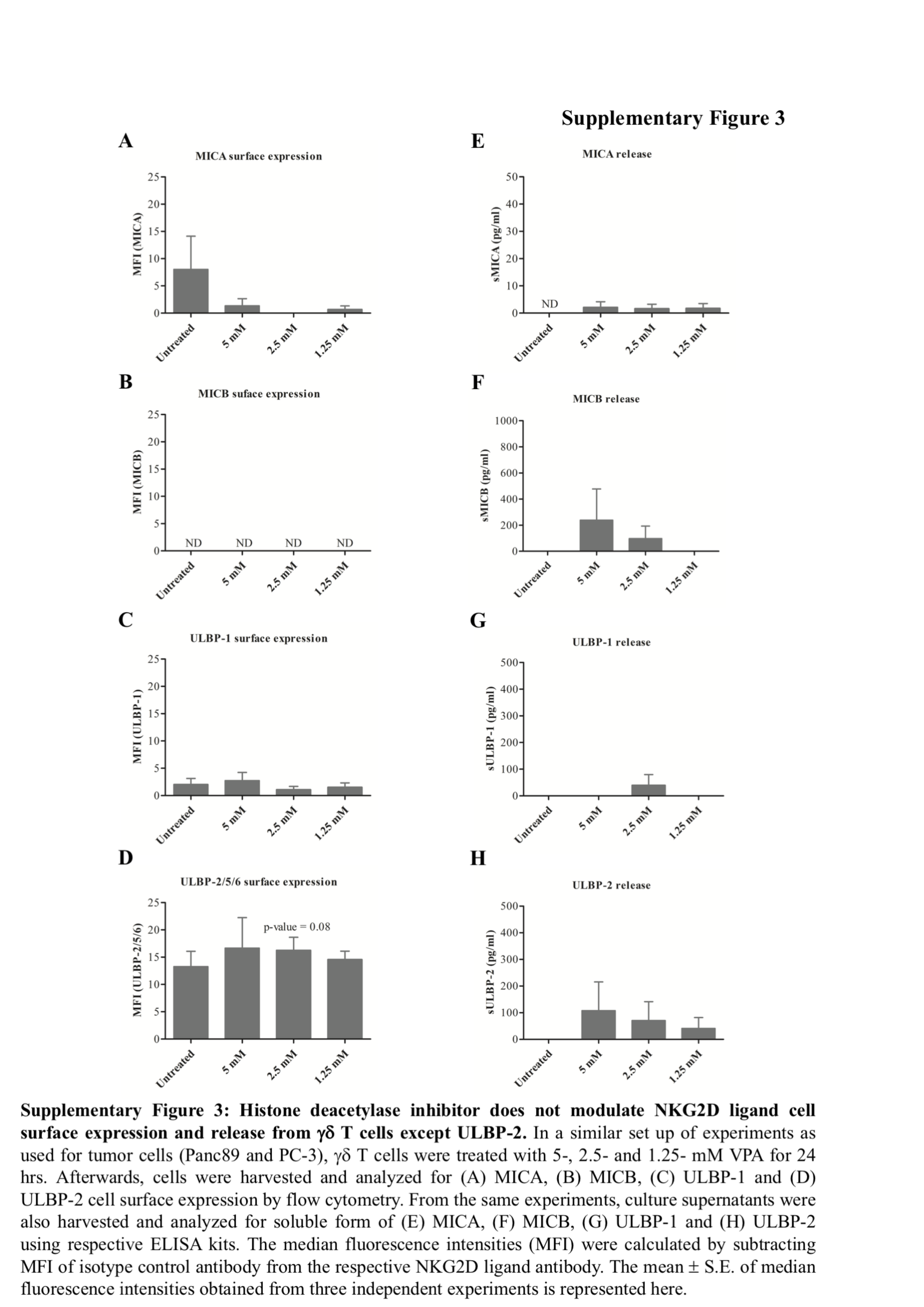

Supplement: Supplementary file 4 [file Image_3.tiff]

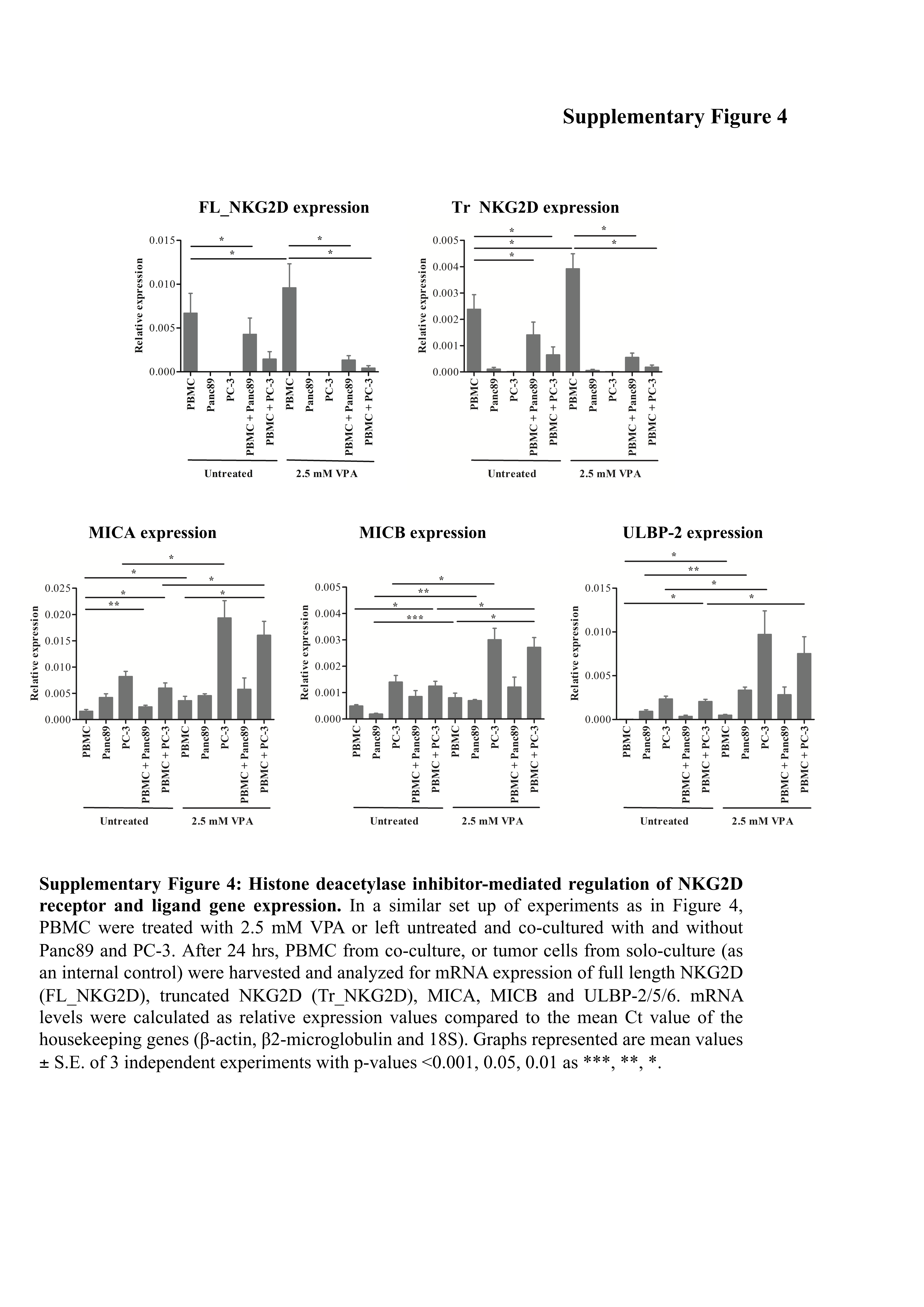

Supplement: Supplementary file 5 [file Image_4.TIFF]

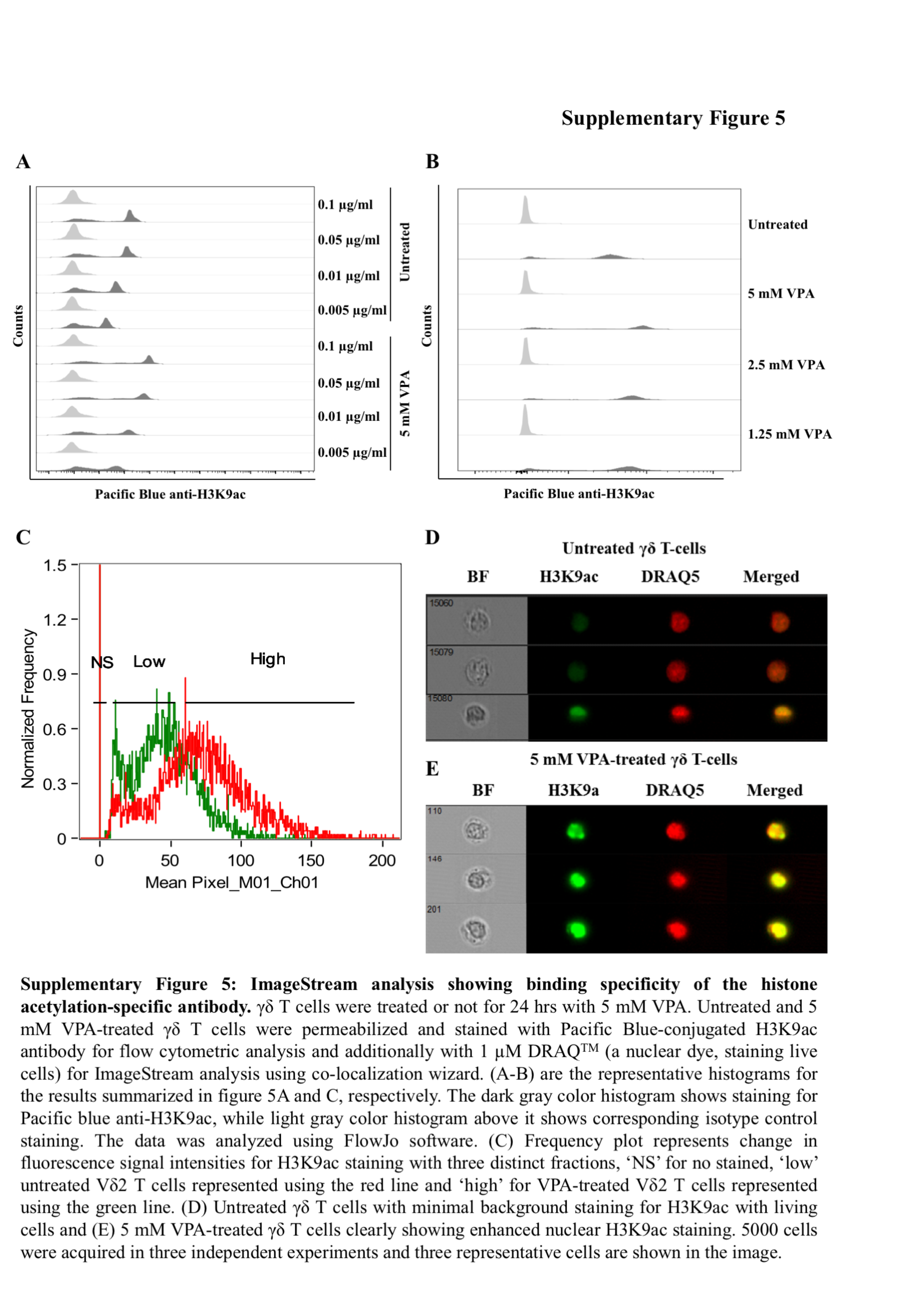

Supplement: Supplementary file 6 [file Image_5.tiff]

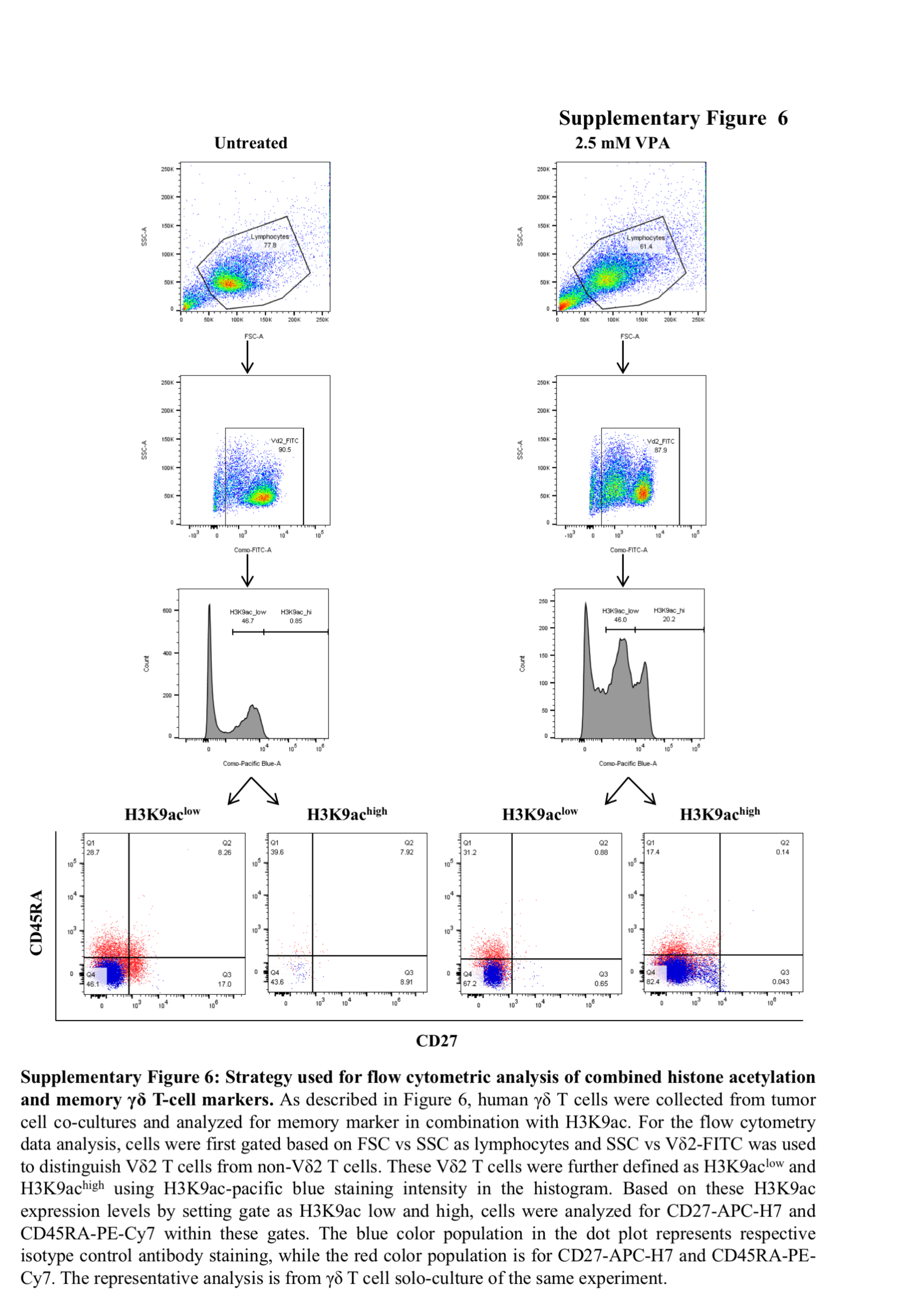

Supplement: Supplementary file 7 [file Image_6.tiff]
